# A network embedding model for pathogenic genes prediction by multi-path random walking on heterogeneous network

**DOI:** 10.1186/s12920-019-0627-z

**Published:** 2019-12-23

**Authors:** Bo Xu, Yu Liu, Shuo Yu, Lei Wang, Jie Dong, Hongfei Lin, Zhihao Yang, Jian Wang, Feng Xia

**Affiliations:** 1 School of Software, Dalian University of Technology, Dalian, 116000 China; 2 Key Laboratory for Ubiquitous Network and Service Software of Liaoning, Dalian, 116000 China; 3 School of Computer Science and Technology, Dalian University of Technology, Dalian, 116000 China

**Keywords:** Prediction of pathogenic genes, Heterogeneous network embedding, Disease-causing genes

## Abstract

**Background:**

Prediction of pathogenic genes is crucial for disease prevention, diagnosis, and treatment. But traditional genetic localization methods are often technique-difficulty and time-consuming. With the development of computer science, computational biology has gradually become one of the main methods for finding candidate pathogenic genes.

**Methods:**

We propose a pathogenic genes prediction method based on network embedding which is called Multipath2vec. Firstly, we construct an heterogeneous network which is called *GP*−network. It is constructed based on three kinds of relationships between genes and phenotypes, including correlations between phenotypes, interactions between genes and known gene-phenotype pairs. Then in order to embedding the network better, we design the multi-path to guide random walk in *GP*−network. The multi-path includes multiple paths between genes and phenotypes which can capture complex structural information of heterogeneous network. Finally, we use the learned vector representation of each phenotype and protein to calculate the similarities and rank according to the similarities between candidate genes and the target phenotype.

**Results:**

We implemented Multipath2vec and four baseline approaches (i.e., CATAPULT, PRINCE, Deepwalk and Metapath2vec) on many-genes gene-phenotype data, single-gene gene-phenotype data and whole gene-phenotype data. Experimental results show that Multipath2vec outperformed the state-of-the-art baselines in pathogenic genes prediction task.

**Conclusions:**

We propose Multipath2vec that can be utilized to predict pathogenic genes and experimental results show the higher accuracy of pathogenic genes prediction.

## Background

Predicting pathogenic genes is important in disease prevention, diagnosis, and treatment [1, 2]. Understanding the pathogenic genes is useful to prevent and control those genetic diseases fundamentally. Revealing the relationship between genetic diseases and disease-causing genes has become an important goal of human genetics [3]. Researchers are committed to predicting the pathogenic genes of diseases and have achieved impressive results [4, 5]. Though some phenotypically similar diseases have been confirmed to be related with some specific genes, many pathogenic genes are still undetected for various reasons. It’s still a great challenge to detect those unknown pathogenic genes.

Traditional genetic localization methods are generally expensive, technique-difficulty and time-consuming. Therefore, there is an urgent need to develop a high-precision method for predicting pathogenic genes [6–8]. It can also improve the efficiency of discovering pathogenic genes and shorten the period of discovering pathogenic genes, laying the foundation for the development of biotechnology, and personalizing gene therapy, etc.

With the accumulation of protein-protein interaction data, it has been a research hotspot in bioinformatics that predicting the pathogenic genes from protein-protein interaction networks [9–12]. Computational biology has gradually become one of the main methods for finding candidate pathogenic genes. Calculating the functional similarity between unknown candidate genes and known pathogenic genes is one of the most popular methods for finding unknown candidate genes. Discovering pathogenic genes by network topological features in human protein-protein interaction networks has made some progress [13, 14]. Moreover, many scholars have made efforts to identify genetic phenotype associations rather than gene-diseases associations [10, 15]. Lage et al. scored protein complexes using gene-phenotype data and genes are ranked according to their asscioation with scored protein complex [10]. Wu et al. established a regression model by calculating a score to measure the correlation between the phenotype similarities and the functional genetic relatedness of disease genes [15].

Some studies have shown that similar phenotypes are generally caused by functionally related genes [16–18]. Driven by this observation, researchers have proposed another method of prediction of pathogenic genes that predict candidate pathogenic genes by gene-phenotype associations [19–22]. Researchers prioritize candidate pathogenic genes of a given disease phenotype by constructing a heterogeneous network that consists of phenotype network, gene(protein) network, and known disease gene-phenotype associations [23]. As it is well known, the phenotypes are regarded as vertices and the links between highly similar phenotypes are regarded as edges in the phenotype network. As for the protein network, the individual proteins are regarded as vertices and the detected protein-protein interactions (PPI) are regarded as edges between the two corresponding vertices. The two networks, i.e., phenotype network and protein network, are connected by the known disease gene-phenotype associations. This kind of heterogeneous network can be used to infer causative genes of a given phenotype. Many methods calculate the similarities between the candidate genes and the target phenotype using the heterogeneous network [24, 25]. Li and Patra proposed a random walk with restart algorithm to infer the gene-phenotype on the heterogenous network [24]. Yang et al. added the information of real protein complexes into the heterogenous network, which constructed a novel protein complex network [25]. However, studies are restricted by the existing big differences between the properties of vertices or links in the heterogenous network. Predicting pathogenic genes of diseases by the heterogenous network is restricted by the complex network properties. Recently in the field of computer science, network embedding algorithms have been proposed [26–30]. Neural network-based learning models can represent latent embeddings into low-dimensional space while capturing the internal relationships of rich and complex data. It has been proved that the network embedding algorithms perform well in clustering, network classification and link prediction, etc [31, 32]. Deep learning techniques are first introduced to analyze graphs in Deepwalk algorithm, which have been proved to be success in natural language processing as well as network analysis [26, 33, 34]. Abundant studies extended and modified the basic Deepwalk model in order to implement this model into the heterogeneous network.

In this work, we use network embedding to predict causative genes in human gene-phenotype heterogeneous networks. We propose a network embedding method called Multipath2vec, which aims to precisely predict pathogenic genes of a target disease. In Multipath2vec, we first construct a human gene-phenotype heterogeneous network. And we design the multi-path which can better capture correlations between different types of vertices to guide random walk in the human gene-phenotype heterogeneous network. Then we use network embedding algorithm to learn features of the constructed networks. Finally, we calculate the similarities between genes and the target phenotypes and then predicts the pathogenic genes. We make the following contributions.

- We propose a pathogenic genes prediction algorithm called Multipath2vec. In Multipath2vec, we propose a special multi-path random walk to make better use of the information of the heterogeneous network.
- We introduce network embedding algorithm in the prediction of pathogenic genes. To our best knowledge, this is the first attempt to exploit the network embedding method in the prediction of pathogenic genes.
- The research strategy of this work can inspire the resolution of analysis task in bioinformatics.

The structure of our paper is organized as follows. “Methods” section illustrates the Multipath2vec algorithm in detail. Experiments are introduced in “Results” and “Discussion” sections concludes the paper.

## Methods

**Fig. 1:**
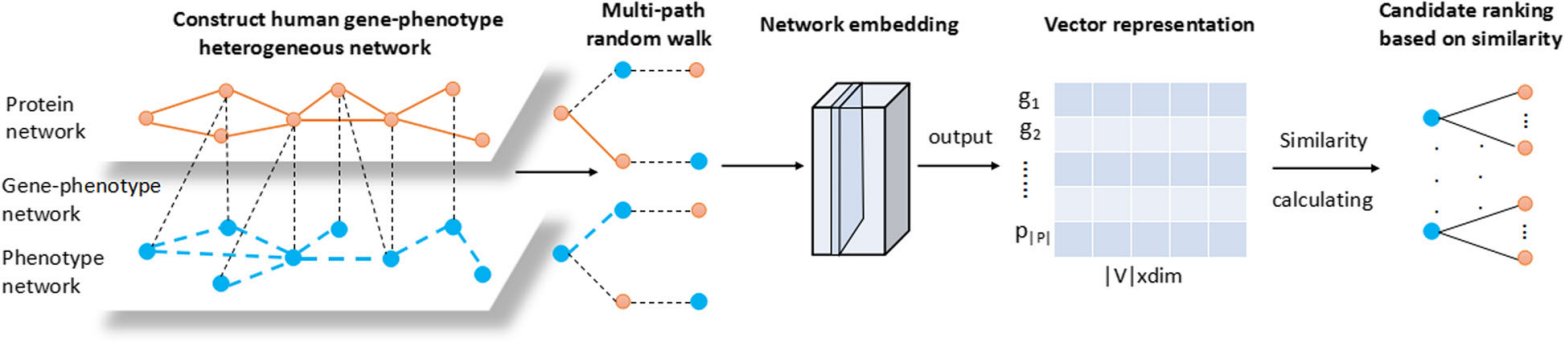
The flow of Multipath2vec. First, we construct the human gene-phenotype heterogeneous network. Based on multi-path guided random walk, we can achieve the vector representation of network according to network embedding. Finally, we calculate the similarities and then rank the candidate genes

In this section, we introduce the detailed description of the construction of the human gene-phenotype heterogeneous network and propose the Multipath2vec algorithm. The flow chart of Multipath2vec is shown in Fig. 1. First, a human gene-phenotype heterogeneous network is constructed based on the correlations between genes and genes, phenotypes and phenotypes, genes and phenotypes. Then we design multi-path to guide random walk in the human gene-phenotype heterogeneous network and represent the network into *d* dimension vectors. And then we calculate the similarities between genes and the target phenotypes. After that, we can get the ranking list of candidate genes.

### Heterogeneous network construction

The heterogeneous network consists of two types of nodes and three types of links. In the heterogeneous network, nodes include the gene nodes and the phenotype nodes. Edges are connected in three relationships: the relationship between phenotype and gene, the relationship between two genes, and the relationship between two phenotypes. The edge between two phenotypes is the link between two highly similar vertices. The edge is connected between two corresponding genes when there exists the experimentally detected protein-protein interaction. Besides, the known disease gene-phenotype associations are used to connect gene and phenotype. For a better understanding, we give the formal definition of the heterogeneous network as follows.

**A Heterogenous Network** is defined as a graph *G*=(*V,E*,*T*) in which each vertex *v* and each edge *e* are associated with their mapping functions *ϕ*(*v*):*V*→*T*_*V*_ and *φ*(*e*):*E*→*T*_*E*_, respectively. *T*_*V*_ and *T*_*E*_ denote the vertex types and relation types, where |*T*_*V*_|+|*T*_*E*_|>2.

For predicting the pathogenic genes of the known disease, we first construct the human gene-phenotype heterogeneous network. In order to precisely describe the relationships in the human gene-phenotype heterogeneous network, we use proteins/genes (i.e., g) and phenotypes (i.e., p) and several relationships between them to represent heterogeneous networks. Proteins/genes and phenotypes are represented as vertices and the edges are denoted as phenotype similarity (i.e., p-p), protein-protein interaction (i.e., g-g), and gene-phenotype association (i.e., g-p/p-g), respectively. We give a clear definition of the human gene-phenotype heterogeneous network that we construct in this paper. We name this network as *GP*−network.

**A*GP*−network** is defined as a graph *G*=(*V,E*,*T*), wherein *V*=*G*∪*P*. *G* is gene set and *P* is phenotype set. *T* is type set, which *T*=*T*_*V*_∪*T*_*E*_. *T*_*V*_ and *T*_*E*_ represents the sets of object type and relation type, where |*T*_*V*_|+|*T*_*E*_|>2. In *G*, each vertex *v* is associated with its mapping function *ϕ*(*v*):*V*→*T*_*V*_ and each edge *e* is associated with its mapping functions *φ*(*e*):*E*→*T*_*E*_.

**Fig. 2:**
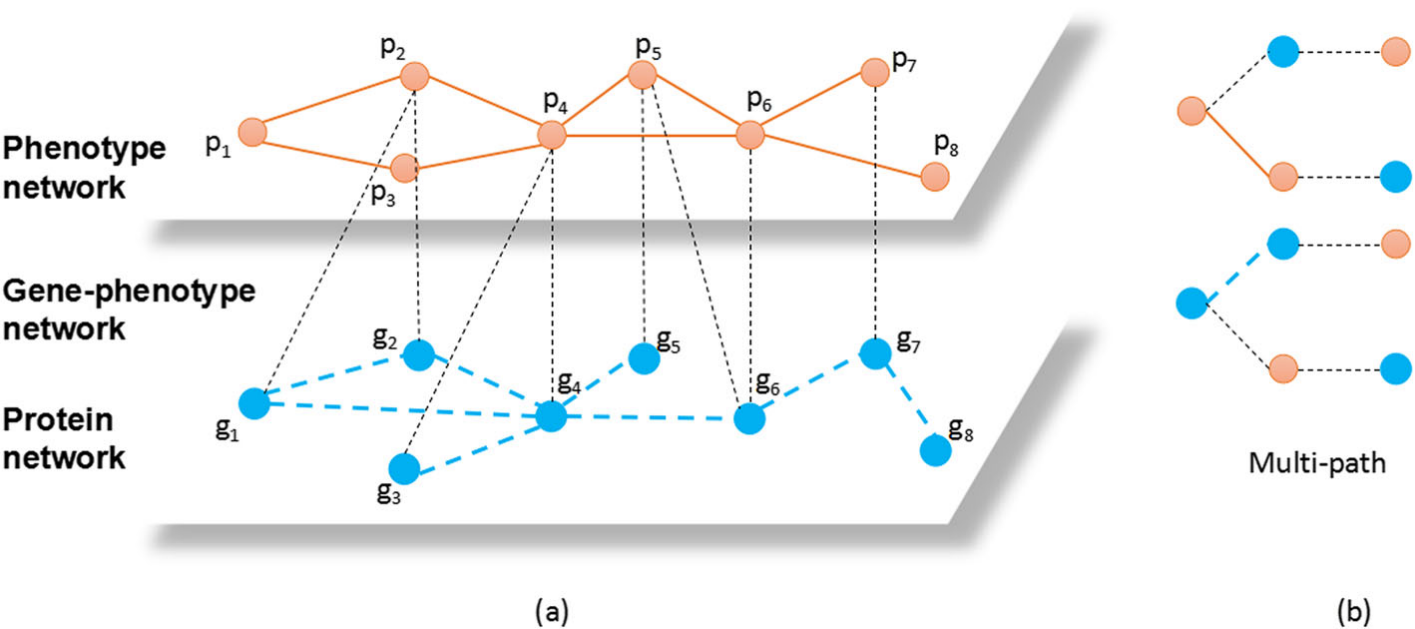
**a** is an example of *GP*−network. Each light orange node represents a phenotype and each blue node represents a protein. The links between phenotypes represent the high similarities. The links between proteins represent the interactions proteins. The black dots represent the associations between genes and phenotypes. **b** is the multi-path used in this work,which is “g-p-g&g-g-p” and “p-g-p&p-p-g”

Figure 2a is an example of *GP*−network. Between genes and phenotypes, there are many associations. Our purpose is to predict the unknown associations between certain genes and phenotypes according to the known links in the *GP*−network.

### Heterogeneous *GP*−network embedding

Dong et al. proposed Metapath2vec, which is a network embedding method for network analysis [35]. In this method, scholars designed meta-path (i.e., a path including different kinds of vertices) to guide random walk [36]. Metapath2vec generates paths through random walks based on meta-path, which can capture rich correlations between different types of vertices. In this paper, we design a novel multi-path to capture richer correlations between vertices. The formal definitions of meta-path and multi-path are respectively introduced as follows.

#### **Definition 1**

In the regular heterogeneous network, a meta-path scheme *H* is defined as a path that is denoted in the form of *V*_1_→*R*_1_*V*_2_→*R*_2_…→*R*_*l*−1_*V*_*l*_, wherein, *R*=*R*_1_◇*R*_2_◇…◇*R*_*l*−1_ defines the composite relations between vertex types *V*_1_ and *V*_*l*_.

A meta-path " *g*−*p*−*g*" represents the common pathogenic genes relationship of a phenotype(*p*) between two genes(*g*). But, the relationship captured by a meta-path is not enough for the heterogeneous network we constructed. For example, if a gene *g*_1_ is the uncovered pathogenic gene of a phenotype *p*_1_, the reasons may be the following two situations: (1) Gene *g*_1_ may interact with *g*_2_, which *g*_2_ has been confirmed to be the pathogenic gene of known phenotype *p*_1_. (2) Gene *g*_1_ may closely associate with *p*_2_ which is highly similar to *p*_1_. Therefore, a meta-path is not suitable for the heterogeneous network we constructed because it can only capture one relationship. Considering this particularity, we propose multi-path based random walk to capture gene-phenotype relationships (*g*−*p*) and gene-gene relationships (*g*−*g*). We define multi-path as follows.

#### **Definition 2**

In *GP*−network, a multi-path scheme *W* is defined as a path that is denoted in the form of *V*_1_→*R*_1_*V*_2_→*R*_2_…→*R*_*l*−1_*V*_*l*_. Wherein, *R*=*R*_1_◇*R*_2_◇…◇*R*_*l*−1_ defines the composite relations between vertices. Besides, *V*_*i*+2_∉*P* when *V*_*i*_∈*P*∧*V*_*i*+1_∈*P*. Likewise, *V*_*i*+2_∉*G* when *V*_*i*_∈*G*∧*V*_*i*+1_∈*G*.

That is, there cannot be three successive vertexes that are all of the same type in a multi-path. Multi-path is more suitable for heterogeneous networks than meta-paths because it can capture multiple relationships simultaneously. Take the situation in Fig. 2a as an example, *g*_2_→*p*_2_→*g*_1_ is meta-path. Different from meta-path, multi-path is allowed to contain two relationships simultaneously. For instance, *g*_2_→*p*_2_→*g*_1_ and *g*_2_→*g*_4_→*p*_4_ are multi-paths. Fig. 2b shows the multi-path used in this work.

Here, we describe how multi-path guides random walkers to walk in the heterogeneous network we build. To a multi-path scheme *W*:*V*_1_→*R*_1_*V*_2_→*R*_2_…→*R*_*l*−1_*V*_*l*_, the transition probability at step *i* is defined as shown in Eq. 1.

1 $$ \begin{aligned}  Pr(v^{i+1}|v_{t}^{i},W)=\left\{ \begin{array}{lr} \frac{1}{\left|N_{t+1}(v_{t}^{i})\right|} \, \left(v^{i+1},v_{t}^{i}\right)\in E,\phi(v^{i+1})=t+1 \\ 0 \qquad \qquad \left(v^{i+1},v_{t}^{i}\right)\in E,\phi(v^{i+1})\neq t+1\\ 0 \qquad \qquad \left(v^{i+1},v_{t}^{i}\right)\notin E \end{array} \right. \end{aligned}  $$

Wherein $v_{t}^{i}\in {V_{t}}$ and $N_{t+1}(v_{t}^{i})$ denotes the neighborhood of $v_{t}^{i}$ as well as being the (*t*+1)^*th*^ type of vertices, *ϕ*(*v*^*i*+1^) represent the type of vertex *v*^*i*+1^. That is, the walker will walk through the pre-defined multi-path *W*. The strategy of the multi-path based random walk ensures the four kinds of relationships can be the input of heterogeneous skip-gram model. One of the advantages of multi-path random walk is that it can capture richer structural correlations.

Given *W*={ *v*_1_…*v*_*l*_} with length *l*, a multi-path guided random walk, the vertex embedding function is denoted by *Φ*(·). *Φ*(·) is learned by maximizing the probability, which is the occurrence that the neighborhood vertices of *v*_*i*_ are within *k* window size conditioned on *Φ*(*v*_*i*_). The objective function is shown in Eq. 2.

2 $$  \min_{\Phi}-\log Pr(\{v_{i-k},\ldots,v_{i+k}\} \backslash v_{i}|\Phi(v_{i}))  $$

To effectively maximize the objective function, we approximate the conditional probability by using the independence assumption. The expression is in Eq. 3.

3 $$  Pr(\{v_{i-k},\ldots,v_{i+k}\} \backslash v_{i}|\Phi(v_{i}))=\prod_{j=i-k,j\neq i}^{i+k} Pr(v_{j}|\Phi(v_{i}))  $$

Heterogeneous skip-gram is used to learn effective vertex representations for a heterogeneous network by maximizing the probability of *Pr*(*v*_*j*_|*Φ*(*v*_*i*_)), it assumes the probability of *Pr*(*v*_*j*_|*Φ*(*v*_*i*_)) is related to the type of vertex *v*_*j*_

4 $$ Pr(v_{j}|\Phi(v_{i}))=\frac{e^{\Psi(v_{j})\cdot \Phi(v_{i})}}{\sum_{u\in V}e^{\Psi(u)\cdot \Phi(v_{i})}},v_{j}\in N_{t}(v)  $$

wherein, *N*_*t*_(*v*) denotes the neighborhood of *v* as well as being the *t*^*th*^ type of vertices.

We also used negative sampling to approximate the objective function for efficient optimization.

5 $$ \begin{aligned} O_{ij}=- \log Pr(v_{j}|\Phi(v_{i}))= \log\sigma(\Psi(v_{j})\cdot \Phi(v_{i}))+ \\ \sum_{m=1}^{M} \log\sigma(-\Psi(v_{jm})\cdot \Phi(v_{i})) \end{aligned}  $$

wherein *σ*(·) is the sigmoid function, and *v*_*jm*_ is the *m*^*th*^ negative node sampled for node *v*_*j*_ and *M* is the number of negative samples. Parameters *Φ* and *Ψ* are updated as follows:

6 $$  \Phi =\Phi - \alpha\frac{\partial O_{ij}}{\partial\Phi},\Psi =\Psi - \alpha\frac{\partial O_{ij}}{\partial\Psi}  $$

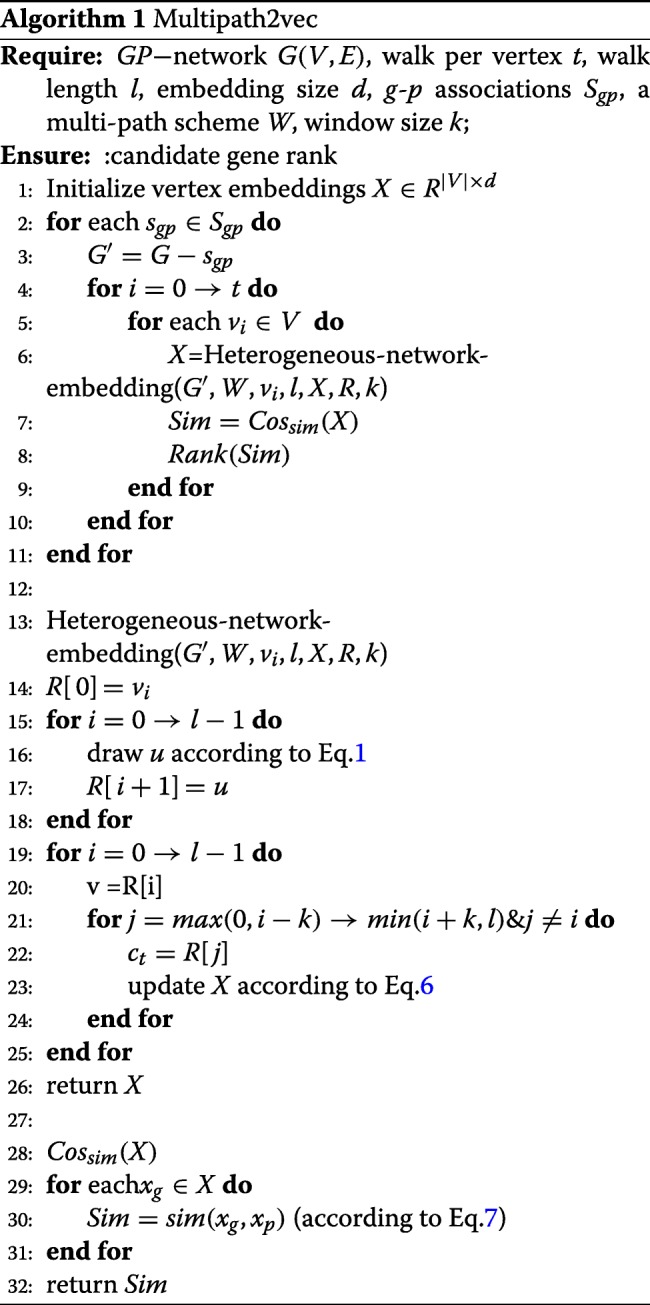


### Score and rank

After getting the vector representation of each phenotype and protein in the human gene-phenotype heterogeneous network, we then calculate the similarity of every gene with the given phenotype. Given a gene *g*=(*x*_1_,*x*_2_,…,*x*_*d*_) and a phenotype *p*=(*y*_1_,*y*_2_,…,*y*_*d*_), we measure the similarity between two vectors using the cosine similarity between the normalized vectors. The calculation formula of similarity is shown in Eq. 7.

7 $$  sim(g,p)=\frac{\sum\limits_{n=1}^{d} x_{n}\ast y_{n}}{\sqrt{\sum\limits_{n=1}^{d} x_{n}^{2}}\ast\sqrt{\sum\limits_{n=1}^{d} y_{n}^{2}}}  $$

After calculating the similarity of every protein in the human gene-phenotype heterogeneous network with the target phenotype, the similarity scores can be ranked in order. Candidate genes are then prioritized. Algorithm 1 shows the whole process of Multipath2vec.

## Results

In this section, we introduce the details about experimental data set, experiment settings, evaluation metrics, baseline approaches and the analysis of experimental results.

### Data sets

We access data sets from three different sources to generate the *GP*−network. The details of these three data sets are described as below.

- **PPI:** We get Human PPI data from the Human Protein Reference database (HPRD). HPRD is a centralized platform which aims at presenting the integrate information about human proteome. The information in HPRD has been extracted by biologists manually. The data set we access from HPRD includes 39,240 interactions among 9,590 human proteins/genes. We filter out the proteins with self-interactions only. After filtering, a total of 8,756 human proteins were used in our experiments.
- **Gene-phenotype associations:** We achieve data of gene-phenotype associations from Online Mendelian Inheritance in Man (OMIM) database. OMIM is an online catalog of human genes and genetic disorders, which focuses on heritable genetic diseases. The data in OMIM includes text information, related reference information, sequence records, maps, and other related databases. In the experiment, we extracted 925 gene-phenotype associations with 667 pathogenic genes and 775 disease phenotypes.
- **Phenotype similarities:** We get the phenotype similarities data from pre-existing research results published in 2006. Van et al.. studied OMIM data base and calculated the similarities [37]. Their research results are uploaded to the website http://www.cmbi.ru.nl/MimMiner/. We use the 5080*5080 phenotype similarity matrix which is calculated by cosine similarity between every two phenotypes.

### Experiment settings

Before generating the *GP*−network, we preprocess the data and set some details in our experiment as follows.

1. Proteins with self-interactions only in the PPI data set are filtered out.
2. In the gene network, we filtered those proteins that are not in gene-phenotype network and also have no links to proteins in the gene-phenotype network. And in the phenotype network, we filtered those phenotypes that are not in gene-phenotype network and also have no links to phenotypes in the gene-phenotype network. In the gene-phenotype network, we filtered those gene-phenotype associations which gene not in the gene network and phenotype not in the phenotype network.
3. In the phenotype network, we connected two phenotypes when their similarity scores are higher than 0.6, which is considered to be reliable according to previous studies [37].

### Evaluation metrics

We used leave-one-out cross validation to verify results in our experiments. Cross validation is also called loop estimation sometimes and is usually used in statistics. It is widely used in the verification of prediction issues. Cross validation can be used to test whether a prediction model is accurate in practical.

#### Leave-one-out cross validation

The first process of cross validation is to separate the original data into two groups, i.e., training set and testing set. Then we use the training set to train the classifier. Finally, we use the testing set to evaluate the classifying quality of classifier. One of the most common used method is leave-one-out cross validation.

In leave-one-out cross validation, leave one sample as testing set and the other samples as training set. We use leave-one-out cross validation in this work since it is suitable for small samples.

#### Precision

Precision is widely used to evaluate the accuracy of prediction. There are four situations in the binary detection, i.e., True Positive (*TP*), True Negative (*TN*), False Positive (*FP*), and False Negative (*FN*). Take Fig. 2 as example, suppose there exist association between gene *g*_10_ and phenotype *p*_8_. If *g*_10_−*p*_8_ is successfully predicted, then this situation is counted as a *TP*. Since the failed prediction is meaningless in this issue, we calculate precision to evaluate the successful prediction rate. The calculation formula is shown in Eq. 8.

8 $$  Precision=\frac{TP}{TP+FP}  $$

### Baseline approaches

We use four methods as baseline approaches, i.e., CATAPULT, PRINCE, Deepwalk and Metapath2vec. We describe the four baseline approaches in detail as below.

1. **CATAPULT:** CATAPULT [38] uses a biased SVM framework and train a bagging support vector machine classifier to classify the gene-phenotype pairs. In CATAPULT, the similarities between vertices can be evaluated by the length of different paths. CATAPULT then uses supervised algorithms to learn the coefficients of different paths. Specifically, the features of gene-phenotype pairs are represented by the number of paths with different lengths in network shown as follows.
  9 $$ C=\left(\begin{array}{cc} N_{GG}&N_{GP}\\ N_{PG}&N_{PP}\\ \end{array} \right)  $$
  wherein, *N*_*GG*_ is the gene network, *N*_*GP*_ is the gene-phenotype network, *N*_*PG*_ is the transposed form of *N*_*GP*_, and *N*_*PP*_ is the phenotype network. By training a bagging classifier, CATAPULT learns the weights of different paths. Then the unconnected paths can be predicted. Considering the negative relationships between vertices do not exist indeed, CATAPULT assumes that the unconnected links are unlabeled and then randomly select some unlabeled relationships as negative association. Therefore, the sample of each SVM classifier are consist of all positive relationships and randomly selected unlabeled relationships. Then we can get a linear classifier *θ*_*t*_. The final results are calculated according to the average value of multiple training models.
2. **PRINCE:** PRINCE is one of the classical algorithms for dealing with this issue. The correlation between the gene *g* and disease *d* is decided by two factors. One is the correlation between neighbor genes of *g* and the target disease *d*. The other one is the priori knowledge of gene *g*. The optimal function of the correlation between the *g* and disease *F*(*g*) is shown as follows.
  10 $$ F(g)=\alpha[\sum_{q\in{N(p)}}wF(p,q)]+(1-\alpha)Y(p)  $$
  Wherein, *w* is normalized form of the weight matrix of the network and *α* is the parameter using to adjust the weight of the two factors.
3. **Deepwalk:** Deepwalk is a network embedding method which is usually used in homogeneous network. Deepwalk learns low-dimensional feature representations by using uniform random walks. It generate random walks by treating nodes of different types equally.
4. **Metapath2vec:** Metapath2vec is proposed for heterogeneous networks. Metapath2vec presented meta-path to guide random walk. Metapath2vec generates paths through random walks based on meta-path, which can capture rich correlations between different types of vertices.

### Experimental results analysis

We use leave-one-out cross validation to evaluate the performance of our method Multipath2vec and four baseline methods in the experiment. We set the experimental parameters as follows.

1. The number of walks per vertex *t*: 500;
2. The walk length *l*: 100;
3. The vector dimension *d*: 128;
4. The neighborhood size *k*: 7;
5. The size of negative samples *M*: 5.

For CATAPULT, PRINCE, Deepwalk and Metapath2vec, we follow the original settings in their previous experiments. In Metapath2vec, we used meta-path " *g*−*p*−*g*". The similarities are calculated through these five approaches and then ranked in the descending order. To better compare these five methods, we calculate the accuracy of Top 1 as well as the lists of Top 5, Top 10, Top 30, Top 50, and Top 100.

Table 1 shows the overall performance of Multipath2vec, CATAPULT, PRINCE, Deepwalk and Metapath2vec approaches on whole gene-phenotype data. We can see that Multipath2vec successfully predicted 317 pathogenic genes at the Top 1 list, whereas CATAPULT, PRINCE, Deepwalk and Metapath2vec successfully predicted 46, 203, 285 and 96 pathogenic genes respectively. As for the Top 5 list, Multipath2vec achieved higher performance with successfully predicting 693 pathogenic genes. Deepwalk predicted 565. Metapath2vec predicted 121. PRINCE predicted 403 and CATAPULT only predicted 57.

**Table 1:** The overall performance of Multipath2vec, CATAPULT, PRINCE, Deepwalk and Metapath2vec methods on whole gene-phenotype data

Algorithm	Multipath2vec	CATAPULT	PRINCE	Deepwalk	Metapath2vec
Top1	317/925	46/925	203/925	285/925	96/925
Top5	693/925	57/925	403/925	565/925	121/925
Top10	793/925	63/925	464/925	669/925	121/925
Top30	860/925	70/925	529/925	790/925	254/925
Top50	878/925	83/925	540/925	824/925	278/925
Top100	897/925	90/925	551/925	859/925	322/925

As for the single-gene gene-phenotype data, the experimental results are shown in Table 2. We can see that Multipath2vec outperforms the other two algorithms. CATAPULT performed worst on single-gene gene-phenotype data. The reason may be that CATAPULT trains a bagging classifier by learning the weights of different paths,but there is only one connected path between target gene and phenotype in single gene data. So we focus on the comparison of Multipath2vec, PRINCE, Deepwalk and Metapath2vec. Multipath2vec successfully predicted 266 pathogenic genes at the Top 1 list, while PRINCE, Deepwalk and Metapath2vec predicted 179, 242 and 48 respectively.

**Table 2:** The overall performance of Multipath2vec, CATAPULT, PRINCE, Deepwalk and Metapath2vec methods on single-gene gene-phenotype data

Algorithm	Multipath2vec	CATAPULT	PRINCE	Deepwalk	Metapath2vec
Top1	266/702	0/702	179/702	242/702	48/702
Top5	532/702	0/702	307/702	491/702	48/702
Top10	606/702	1/702	353/702	538/702	48/702
Top30	651/702	5/702	405/702	596/702	102/702
Top50	662/702	16/702	411/702	618/702	110/702
Top100	678/702	23/702	417/702	644/702	137/702

Moreover, we list the overall performance of these five methods on many-genes gene-phenotype data in Table 3. As shown in the Table 3, Multipath2vec still outperforms.

**Table 3:** The overall performance of Multipath2vec, CATAPULT, PRINCE, Deepwalk and Metapath2vec methods on many-genes gene-phenotype data

Algorithm	Multipath2vec	CATAPULT	PRINCE	Deepwalk	Metapath2vec
Top1	51/223	46/223	24/223	43/223	48/223
Top5	161/223	57/223	96/223	74/223	73/223
Top10	187/223	62/223	111/223	131/223	73/223
Top30	209/223	65/223	124/223	194/223	152/223
Top50	216/223	67/223	129/223	206/223	168/223
Top100	219/223	67/223	134/223	215/223	185/223

**Fig. 3:**
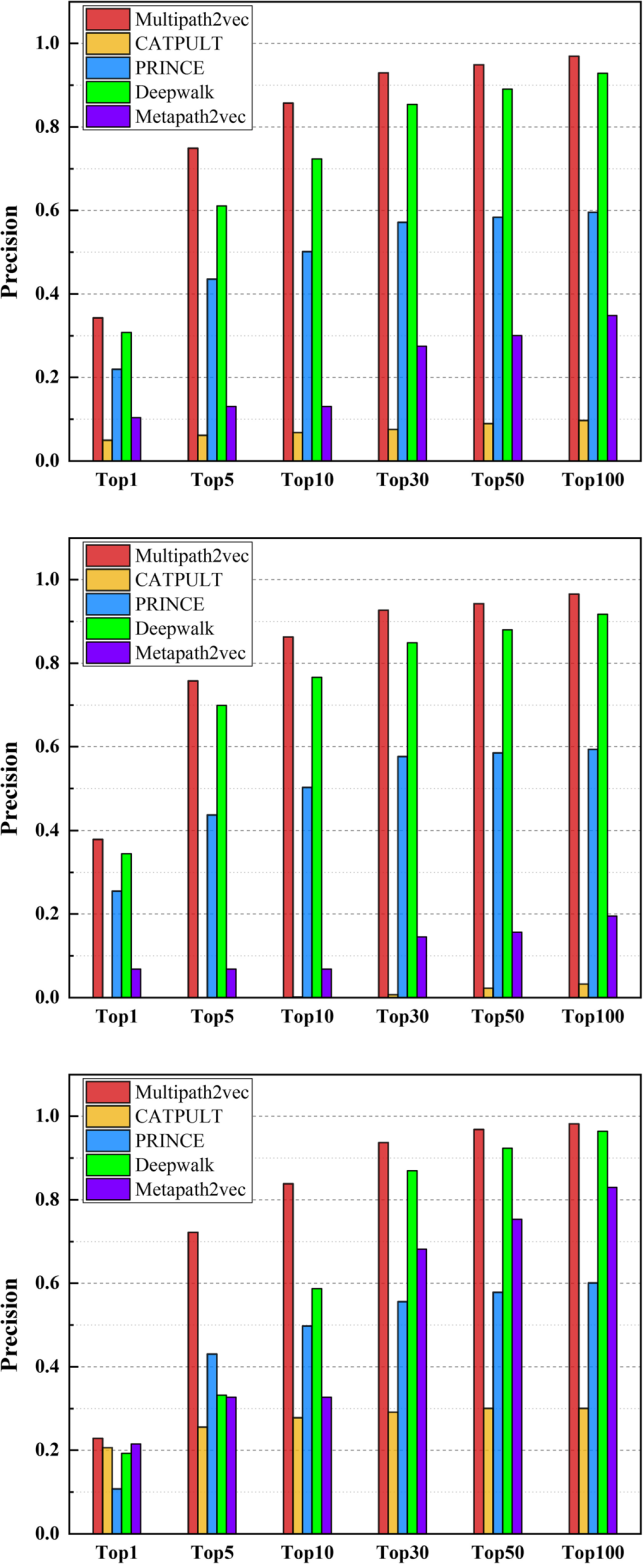
The precision values of Multipath2vec, CATAPULT, PRINCE, Deepwalk and Metapath2vec, grouping by Top 1, 10, 30, 50, 100 on whole-genes gene-phenotype data,one-gene gene-phenotype data and many-genes gene-phenotype data,respectively

**Fig. 4:**
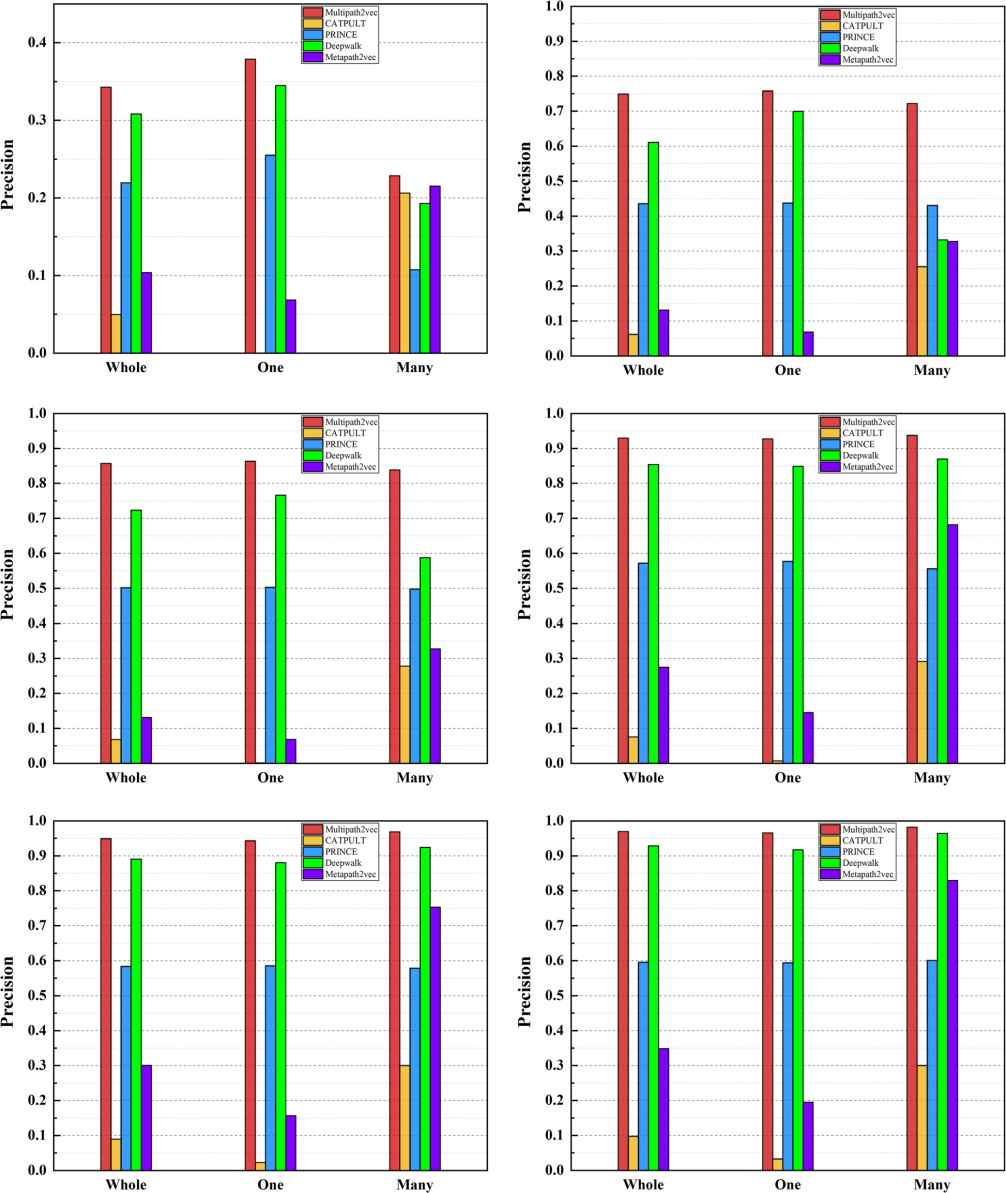
The precision values of Multipath2vec, CATAPULT, PRINCE, Deepwalk and Metapath2vec on Top 1,Top 5,Top 10,Top 30,Top 50 and Top 100 evaluation, grouping by whole-genes, single-genes, and many-genes gene-phenotype data, respectively

We also calculate the precision values of these five methods, which are shown in Figs. 3 and 4, respectively. Figure 3 shows the precision values of the five methods under the 6 different groups on single-genes, many-genes and whole-genes gene-phenotype data, respectively. We choose 6 groups of different sizes as mentioned above, i.e., Top 1, Top 5, Top 10, Top 30, Top 50, and Top 100. It can be obviously seen from Fig. 3 that the precision values of Multipath2vec outperform CATAPULT, PRINCE, Deepwalk and Metapath2vec.

Figure 4 shows the precision values of Multipath2vec, CATAPULT, PRINCE, Deepwalk and Metapath2vec, grouping by single-genes, many-genes and whole-genes gene-phenotype data, respectively. In general, Multipath2vec outperforms the other four approaches. The performance of Multipath2vec and Deepwalk are ahead of the other baseline approaches. Deepwalk performs closely with Multipath2vec but still cannot catch up with Multipath2vec. Wherein, CATAPULT performs worst so that it cannot compare with Multipath2vec, PRINCE, Deepwalk and Metapath2vec.

In summary, Multipath2vec can successfully predict pathogenic genes with high accuracy. The experimental results shows that Multipath2vec outperformed baseline approaches in all perspectives. Therefore, Multipath2vec is able to be used in predicting pathogenic genes.

## Discussion

### Robustness of false negative

In our experiment, we used precision to evaluate the accuracy of prediction. In each round of leave-one-out cross validation, if the cut link between the target gene and phenotype is successfully predicted, then this situation is counted as a *TP*. And we can also regard this situation as a *TN* because the negative is successfully predicted as a negative. So the number of *TP* is equal to the number of *TN* and the number of *FP* is equal to the number of *FN*. Our method perform well in the accuracy of prediction, so it is also robust to false negative.

## Conclusion

The study of pathogenic genes plays an important role in revealing the pathogenesis of diseases as well as developing corresponding disease prevention and diagnosis methods. The key to deciphering the molecular and genetic basis of human disease is to analyze the correlation between diseases and genes. In this paper, we propose the Multipath2vec algorithm which is based on network embedding to predict pathogenic genes. The multi-path in Multipath2vec are designed to guide random walk in the human gene-phenotype heterogeneous network. The multi-path based random walk can better represent the network. The experimental results show that Multipath2vec outperforms four baseline methods from several perspectives. By implementing these three approaches on single-gene gene-phenotype data, many-genes gene-phenotype data and whole-genes gene-phenotype data, Multipath2vec showed the outstanding performance in prediction of pathogenic genes. By calculating the precision values of these five methods, Multipath2vec still outperforms under all circumstances. This fact illustrates the possibility of applying heterogeneous network embedding approach in prediction of pathogenic genes.

## Data Availability

The vectors trained during the current study are available from the corresponding author on reasonable request.

## Abbreviations

*GP*−network: The gene-phenotype network
FN: False negative
FP: False positive
HPRD: The human protein reference database
OMIM: Online mendelian inheritance in man
PPI: Protein-protein interaction
TN: True negative
TP: True positive
